# One Obstacle to Social Reform

**Published:** 1913-12

**Authors:** 


### One Obstacle to Social Reform

An enlightening interview occurred recently, worth being considered seriously, although narrated as amusing. A prominent physician, unusually religious as medical men go, and unusually moral as men in general run, consulted the chief of police of a large city in regard to the elimination of houses of prostitution. "Well, you see, Doc, you and I have had ours . . . ." was the gist of the explanation why the task of cleaning out these stables could not be accomplished. There, in our opinion, is the crux of the whole situation. Speaking in average terms, the police officer, his superiors, the judge, the juryman, the attorneys, and the citizen who creates public sentiment, which is the backing of all enforcement of law, as well as the legislator who makes the law and the executive who signs it, has had or is having his. The whole machinery by which the elimination of the house of prostitution or any other social reform is to be accomplished depends upon energy obtained from firing a mixture of ingredients. These consist of the influence (1) of the morally pure, (2) of the sexually weak, (3) of the morally impure who have reformed or who have become sexually weak from normal or premature senility, (4) of the actively immoral class. We do not pretend to estimate the proportions of these various classes save to hold that the first two classes are small and the fourth quite large. What we do want to emphasize is that the third and fourth classes can lend to the reform movement no strength, except that of the hypocrites who, having had or still enjoying theirs, have no objection to gaining favor by treating less discreet persons as criminals. And the strength due to hypocrisy is weakness. When the majority of healthy, virile men have grown up as a moral generation, they can and will, consistently demand the same standard of public virtue. But no dependence can be placed on legislation and inforcement of laws against prostitution from men who have had theirs.

